# The Effect of a Mandatory Play Break on Subsequent Gambling Behavior among British Online Casino Players: A Large-Scale Real-World Study

**DOI:** 10.1007/s10899-022-10113-x

**Published:** 2022-03-15

**Authors:** Michael Auer, Mark D. Griffiths

**Affiliations:** 1 neccton GmbH, Davidgasse 5, 7052 Muellendorf, Austria; 2 International Gaming Research Unit, Psychology Department, Nottingham Trent University, 50 Shakespeare Street, NG1 4FQ Nottingham, UK

**Keywords:** Gambling, Responsible gambling, Responsible gambling tools, Problem gambling, Mandatory play breaks, Forced session termination

## Abstract

In recent years, various novel responsible gambling (RG) tools have been implemented to aid harm-minimization. One such RG tool has been the implementation of enforced mandatory play breaks. Despite many responsible gambling operators using mandatory play breaks, only three previous studies have examined their efficacy and the findings were mixed. Therefore, the present investigation was a large-scale real-world study which was designed to see whether a 60-minute mandatory play break influenced subsequent depositing and wagering. The authors were given access to 27 days of player data prior to the introduction of a mandatory play break and 27 days of player data after the mandatory play break was introduced. The study comprised British online gamblers from *Skillonnet* (a European online gambling operator). Between July 23 and September 15 (2021), 2,021 players deposited at least ten times or more on a calendar day, at least once. The 2,201 players generated 2,994 corresponding events (i.e., the depositing of money at least 10 times in one day). The percentage of players who stopped depositing money as a consequence of the mandatory play break rose from 27% to 68% on the day of a play break. Moreover, the percentage of players who stopped wagering as a consequence of the mandatory play break rose from 0.1% to 45% on the day of a play break. The findings of the present study demonstrated that a 60-minute mandatory play break impacts players’ depositing and wagering immediately after the play break. This means that a mandatory hour-long play break in an online casino setting appears to prevent overspending during a short period of time. The effects of a 60-minute mandatory break on the next day’s behavior were inconclusive.

## Introduction

In recent years, the improved coverage, convenience, and widespread availability of the internet have been facilitating factors for the increased popularity of online gambling. Generally, online gambling is less popular than land-based gambling. However, in recent years online gambling has become more popular, especially among younger people (Gómez et al., 2019; Hollén et al., 2020; Molinaro et al., 2018). Moreover, online gambling has been associated with problem and pathological gambling among a minority of individuals (Lawn et al., 2020). Several studies report that online gamblers are more likely to show signs of problematic gambling than those who do not gamble online (e.g., Chóliz et al., 2019; Effertz et al., 2018; Griffiths et al., 2009; Volberg et al., 2018) although most online gamblers also gamble in offline environments too (Wardle et al., 2011).

In online gambling, each transaction is assigned to an individual player account which means that various novel responsible gambling (RG) tools can be implemented to aid harm-minimization. Such strategies include the use of mandatory breaks in play, ‘pop-up’ messaging, personalized messaging. limit-setting, and behavioral tracking tools (Harris & Griffiths, 2017). Various experimental and real-world studies have shown that voluntary limit-setting can reduce subsequent gambling losses (e.g., Auer & Griffiths 2013; Auer, Hopgartner & Griffiths, 2020; Wohl et al., 2013; Wohl et al., 2014). Pop-up messages which appear while players are gambling have also been subject to several studies (e.g., Auer & Griffiths 2015; Auer et al., 2014). Auer et al., (2014) reported in a study of approximately 50,000 online gamblers that less than 1% of players who received a simple pop-up message after they had played 1,000 consecutive slot games stopped playing. In a follow-up study, Auer and Griffiths (2016) redesigned the pop-up message and incorporated normative feedback and a recommendation to set limits. The study found that the new ‘enhanced’ pop-message increased the efficacy compared to the simple message with the number of sessions ceasing after the pop-message being more than doubled. However, the absolute number of players who stopped gambling as a consequence of the pop-up message was still very low (less than 2%).

Contrary to voluntary measures, some countries (like Norway) have introduced mandatory loss limits (Auer et al., 2020). Based on evidence from peer-reviewed and grey literature, Delfabbro and King (2021) compared the efficacy of voluntary vs. mandatory limit-setting. They concluded that relatively few gamblers used voluntary limit-setting technologies and that limit-setting generally had only a modest impact on gambling behavior in reducing expenditure. However, they also concluded that mandatory limit-setting systems in Norway showed promising results. Moreover, it should be noted that implementing such expenditure restrictions could lead to higher risk gamblers migrating elsewhere to potentially more harmful and less regulated operators.

Mandatory play breaks are another type of RG tool that online operators can use to aid harm-minimization. Here, online operators can block players from gambling for a short period of time after they have displayed excessive gambling behavior (e.g., engaging in a long play session, experiencing high monetary losses, frequently making monetary deposits, etc.). McAuliffe et al. (2021) conducted a review of 86 studies regarding responsible product design to mitigate excessive gambling. They concluded that the product safety literature provided the best evidence for pop-ups with self-appraisal messaging, breaks in between rounds of play, precommitment to less risky bets, removal of electronic gaming machine (EGM) features that promote excessive gambling, providing recommendations that minimize house edge, and removing banknote acceptors. It has been argued that long playing sessions can create a dissociative state which in turn could lead to overspending (Griffiths et al., 2006). Jacobs( 1988) found dissociative states to be common among addicts (including problem gamblers). In a review concerning the understanding of slot machine behavior, Murch and Clark (2021) outlined the importance of dissociative experiences which are correlated with gambling problems and may be amplified by specific features of EGMs and other modern gambling formats. It is assumed that mandatory play breaks can help in the termination of a potentially dissociative state and avoid excessive losses. Despite the introduction of mandatory play breaks by many responsible gambling operators, evidence for their efficacy is limited.

The first study examining the efficacy of mandatory play breaks was an experimental laboratory-based study by Blaszczynski et al. (2015). Their experiment examined three different play break conditions (no break in play, a three-minute play break, and an eight-minute play break) and their perceived impact on cravings for gambling (using a gambling craving scale). The study comprised 141 participants (all university students; 63 males) all of who played simulated electronic blackjack for a 15-minute period. The findings indicated participants in the longest (eight-minute) play break condition reported significantly higher craving than those in the other two conditions. Self-reported craving was also higher among participants in the three-minute play break condition than those who had no play break. There were no significant differences among participants’ levels of self-reported dissociation. These findings suggest that forced play breaks may have some unintended consequences (i.e., increased craving which may result in individuals continuing gambling rather than curtailing it). However, the study was small-scale, lacked ecological validity, and the period of gambling prior to the forced play break was only a quarter of an hour.

More recently, two real-world studies have investigated the effects of mandatory play breaks with actual players on video lottery terminals (VLT) and a real gambling website. Auer et al.(2019a) studied 7,190 Norwegian video lottery terminal (VLT) players who experienced a forced session termination of 90 s after a one-hour play duration on the *Norsk Tipping* VLTs. The control group was based on a matched pairs design of similar players who played for slightly less than one hour and therefore did not experience an enforced play break. Results demonstrated that there was no significant effect of the forced termination regarding the amount of money staked in the subsequent gambling session or on the time duration of the subsequent gambling session. The results also indicated that forced terminations led to higher expenditure in the subsequent 24 h among those gamblers who had been forced to take a play break. However, the study lacked a proper control group as none of the matched pairs exceeded gambling for a one-hour period.

In a real-world experimental study by Hopfgartner et al. (2021), 21,129 online gamblers of Norwegian gambling operator *Norsk Tipping* were assigned to one of eight experimental groups. The groups differed with respect to a number of criteria, one of them being the length of a mandatory play break after one hour of consecutive play. That study investigated the differences between a 90-second, five-minute and 15-minute mandatory play break after one hour of consecutive play. The results indicated that a 15-minute mandatory play break led to a disproportionately longer voluntary play pause following the mandatory play break compared to 5-minute and 90-second mandatory play breaks. The median voluntary play pause after the play break for players whose gambling was interrupted for five minutes or 90 s was less than two minutes. If the mandatory play break was 15 min, the median play pause was more than six minutes.

Given the mixed findings concerning the efficacy of mandatory play breaks in the few previous studies, the present investigation was a large-scale real-world study which was designed to answer the following research questions (RQs):

- RQ1: Does a 60-minute mandatory play break influence subsequent depositing and wagering on the day of the play break?
- RQ2: Does a 60-minute mandatory play break influence subsequent depositing and wagering on the next day?
- RQ3: Is there a correlation between pre-play break money lost and post-play break depositing or wagering? It was posited that craving could be the reason for more wagering/depositing after the play break if the losses were larger than expected before the play break.
- RQ4: Is there a correlation between the amount of money won prior to the 60-minute mandatory play break and depositing as well as wagering after the play break?
- RQ5: Does gambling intensity change over time as a consequence of the introduction of a 60-minute mandatory play break?
- RQ6: Does a 60-minute mandatory play break have an impact on loyalty?

The answers to these research questions are likely to have important impacts on technical possibilities in the prevention of gambling disorder.

## Method

### Study context

The present study was carried out comprising British online players from *Skillonnet* (a European online gambling operator). *Skillonnet* offers a variety of different games including slots, roulette, blackjack, baccarat, live-casino games, and live roulette. *Skillonnet* provided anonymized player tracking data from several UK-based online casino sites for secondary analysis (i.e., analysis on a pre-existing dataset). Player tracking data were provided for the time period between July 23 and September 15, 2021. On August 20, 2021, *Skillonnet* introduced a mandatory 60-minute play break if players made ten deposits into a gambling account on a single calendar day. The timestamp of each deposit was available for the entire observation period from July 23 to September 15. The first, second, third, and tenth deposit timestamp could be computed from the available raw data. The authors chose a control period from July 23 and August 19 and a treatment period from August 20 and September 15. The 27 days in each period were chosen because there is a strong periodicity with respect to weekdays. Fridays and Sundays are the days that have the largest number of active players and the remaining five days have the lowest gambling activity. The 27-day control period and the 27-day treatment period have the exact same number of Sundays, Mondays, Tuesdays, etc.

**Fig. 1:**
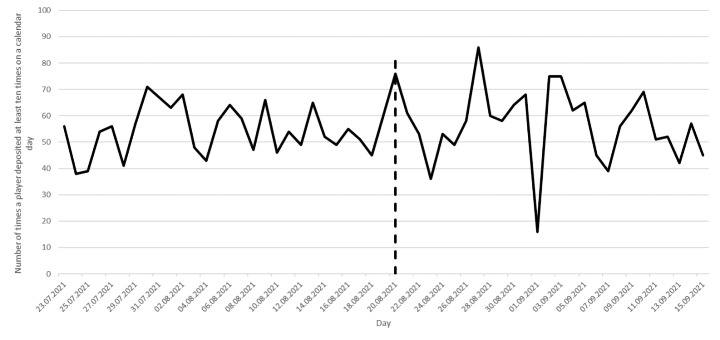
Number of times players deposited money at least 10 times for each day between July 20 and September 15. The dashed vertical line separates the control and treatment period

Between July 23 and August 19 (the control period), there was no mandatory play break after the tenth deposit. Players could deposit and play in the 60 min after the tenth deposit as often as they wanted. Between August 20 and September 15 (the treatment period), players could not play or deposit for 60 min starting with the exact timestamp of the tenth deposit on a calendar day. After the 60 min elapsed, they could deposit and play again if they wanted to do so. Figure 1 displays the number of times players deposited at least ten times in the control period and the treatment period. There were 1,461 events (i.e., the depositing of money at least 10 times in one day) in the control period and 1,533 in the treatment period. The dashed vertical line separates the control period and the treatment period. No major changes or promotions happened on the *Skillonnet* site during the study period.

### Statistical analysis

The authors were given access to a *Skillonnet* secondary dataset which comprised each bet and each win, as well as each deposit and each withdrawal. Appendix 1 reports the variables which were computed based on the raw data provided by the gaming operator. The amount of money deposited, withdrawn, wagered, won, lost, number of games, and number of deposits were computed for the session during which the tenth deposit occurred. Moreover, the amount of money which was in the gambling account (last balance) when the session ended as well as the session length in minutes were also computed.

Sessions were computed based on the timestamp of the single wagers. If two wagers were placed within 15 min of each other, the time between those two events counted as session time. If there was more than 15 min between two wagers, the time between the two events was not counted as session time. Several other research studies have used 15 min to define session length (e.g., Auer et al., 2019a) so this convention was followed. The authors were also aware that most gambling operators use 15 min to compute session time. The amount of money deposited and wagered from 00:00 until the tenth deposit were also computed. The same two metrics were computed for the next calendar day if a player had deposited money at least ten times on the previous day.

Mann-Whitney U-Tests (Mann & Whitney, 1947) were used due to the skewed distribution of the amount of money wagered, lost, won, withdrawn, and deposited. The Shapiro-Wilk test was used to test for a normal distribution (Razali et al., 2011). A logistic regression was applied on the data from the treatment period. The binary dependent variable indicated whether players deposited until midnight after a mandatory play break and the independent variables were the session metrics as well as the behavior from midnight until the mandatory play break.

### Participants

Between July 23 and September 15 (2021), 2,021 players deposited at least ten times or more on a calendar day, at least once. The players’ average age was 38.20 years (*SD* = 10.77), and 1,105 players were female (55%) and 916 players were male (46%). The 2,021 players produced 2,994, events. An ‘event’ refers to a player depositing at least ten times during one calendar day. A total of 1,461 events occurred between July 23 and August 18 (control period) and 1,533 events occurred between August 20 and September 15 (treatment period). August 19 was excluded from the analysis because the mandatory play break was introduced by the gaming operator on that day. However, the authors did not know the exact time when the mandatory play break was introduced on that day so data from that day were excluded from the analysis.

## Results

### Demographic statistics

The average age of players who deposited at least once ten times or more on a calendar day in the control period was 38.28 years (SD = 10.74) and 56% were female. This compared to 38.24 years (SD = 10.70) and 54% female in the treatment period. There was no significant difference with respect to age (*t* = 0.077, *p* = 0.94) or gender (z=-1.51, *p* = 0.13) between participants in the control period and the treatment period.

### Data distribution statistics

There was a significant deviation from a normal distribution for the amount deposited since midnight before the tenth deposit in the control period using the Shapiro-Wilk test (W = 0.51, *p* < 0.001). There was no significant difference in the amount of money deposited from midnight before the tenth deposit between the control period and the treatment period using the Mann-Whitney U-test (U = 1,102,408, *p* = 0.46). There was a significant deviation from a normal distribution for the amount of money lost from midnight before the tenth deposit in the control period using the Shapiro-Wilk test (W = 0.700, *p* < 0.001). There was no significant difference in the amount of money lost from midnight before the tenth deposit between the control period and the treatment period using the Mann-Whitney U-Test (U = 1,100,804, *p* = 0.42).

### Mandatory play break efficacy on the day of the play break (RQ1)

**Fig. 2:**
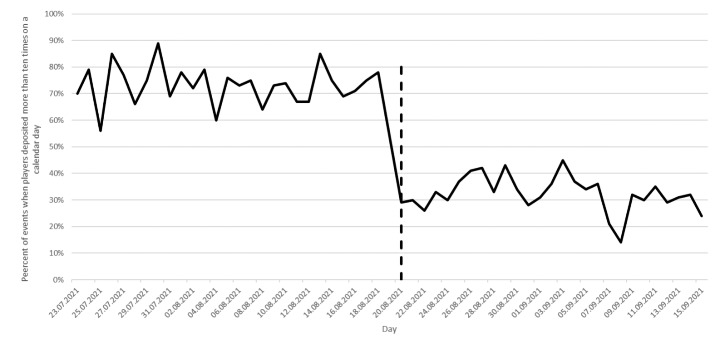
Percentage of events when players deposited more than 10 times on a calendar day. The denominator for the percentage computation is displayed in Fig. 1

In order to evaluate the effectiveness of the 60-minute mandatory play break, the authors computed the number of players who deposited more than ten times during the control period and the treatment period. During the control period, players could deposit immediately after the tenth deposit and in the treatment period there was a 60-minute time window during which players could not play or deposit. Figure 2 displays the percentage of events when players deposited more than ten times on a calendar day. Figure 1 shows that on July 23, 56 players deposited at least ten times. Figure 2 shows that of these 56 players, 39 deposited at least eleven times on that day (70%). Seventeen players stopped depositing after the tenth deposit on that calendar day (30%).

On August 20, the first day after the introduction of the mandatory play break, 76 players deposited at least ten times. Of these, 76, 22 deposited at least eleven times (29%) and 54 players stopped depositing after the tenth deposit on that calendar day (61%). On average during the control period, 73% of players deposited at least eleven times from the players who deposited at least ten times. On average during the treatment period, 32% of players deposited at least eleven times from the players who deposited at least ten times. This difference was statistically significant (*t* = 21.43, *p* < 0.0001). During the control period, 99.9% of players wagered at least once after then tenth deposit. During the treatment period, 55% of players wagered at least once after the tenth deposit. This difference was statistically significant (*t* = 29.33, *p* < 0.0001).

### Mandatory play break efficacy on the day after the play break (RQ2)

**Fig. 3:**
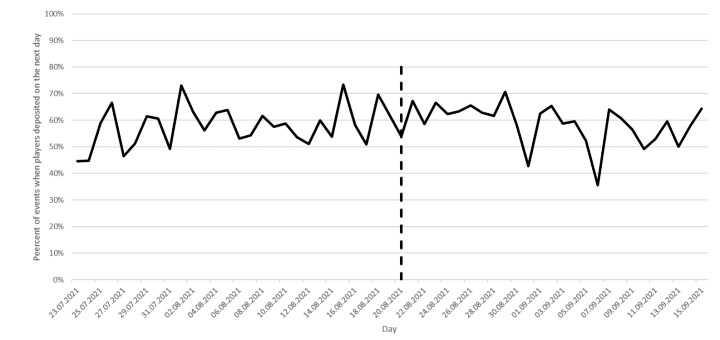
Percentage of events when players deposited on the next day, given they deposited at least ten times on the previous day

The depositing of money the following day was another metric used to evaluate the effectiveness of the mandatory play break. Figure 3 shows the percentage of events when players deposited money on the next day. In the control group, on average 58% of players who deposited money at least ten times during a single day, deposited on the next day. During the treatment period, on average 59% of players who deposited money at least ten times during a single day, deposited on the next day. This difference was not statistically significant (*t*=-1.16, *p* = 0.25). In the control group, on average 66% of players who had deposited money at least ten times during a single day, wagered on the next day. In the treatment group, on average 71% of players who had deposited money at least ten times during a single day, wagered on the next day. This difference was statistically significant (*t*=-2.134, *p* = 0.023).

### Losing/winning and depositing propensity (RQ3 and RQ4)

The authors also analyzed whether there was a correlation between losing/winning and the propensity to deposit money on the same day after a mandatory play break as well as the day after a mandatory play break. The 1,533 events during the treatment period were classified into ten groups according to the amount of money lost in the session prior to the mandatory play break. All players in Groups 1 to 9 lost. This means that their amount wagered was larger than the amount won. Group 10 contains the players who won more than they wagered. Among the players with the highest monetary losses (Group 1), 36% deposited money at least once more on the same day after the mandatory play break and 45% in this group deposited money on the next day. Among the players who won more money than they wagered (Group 10), 39% deposited money at least once more on the same day after the mandatory play break and 69% in this group deposited money on the next day. No significant difference was found with respect to depositing money the same day after a mandatory play break across the ten groups (χ^2^ = 8.27, *p* = 0.49). A significant difference was found with respect to depositing money the next day after a mandatory play break across the ten groups (χ^2^ = 27.66, *p* = 0.001).

### Regression analysis

Logistic regression analysis was performed on the data from the treatment period. The binary dependent variable indicated whether players deposited up to midnight following a mandatory play break and the independent variables were the session metrics as well as the behavior from midnight until the mandatory play break. A Nagelkerke R^2^ of 0.023 indicated very low model quality (i.e., only 2.3% of the variance was explained by the independent variables). Three *p*-values were smaller than 0.05: (i) the session before the mandatory play break occurred between 01:00am and 05:00am (i.e., those who received a mandatory play break between 1am-5am were more likely to deposit money again at least once during the calendar day); (ii) the amount of money won in the session before the mandatory play break (i.e., the larger the amount of money paid back as winnings in the session before the mandatory play break, the less likely the player was to deposit money again at least once during the calendar day); and (iii) the maximum amount of money in the player’s account during the session before the mandatory play break (i.e., the larger the maximum amount of money a player had in the gambling account during the session before the mandatory play break, the more likely the player was to deposit money again at least once during the calendar day).

### Mandatory play breaks and gambling intensity over time (RQ5)

The authors also evaluated whether the introduction of a 60-minute mandatory play break resulted in a change of gambling intensity over a longer period of time. Therefore, the number of players who received at least one mandatory play break at the start of the treatment period between August 20 and August 26 was computed. A total of 333 players experienced at least one mandatory play break during these seven days. For these 333 players, the amount of money deposited, amount of money wagered, and session length during the past seven days of the treatment period between September 9 and September 15 was computed. The same procedure was applied to the control period. In the control period the seven-day period was July 23 to July 29. Here, 301 players deposited money at least on one day ten times during these seven days. For these 301 players, the amount of money deposited, amount of money wagered, and session length were computed for the last seven days of the control period between August 12 and August 18. There were no significant differences in the amount of money deposited (U = 9729, p = 0.68), amount of money wagered (U = 9217, *p* = 0.71) or session length (U = 9386, p = 0.91) between the control period and the treatment period.

### Mandatory play breaks and loyalty (RQ6)

The authors also evaluated whether the introduction of a 60-minute mandatory play break had an impact on loyalty. Therefore, the number of players who received at least one play break at the beginning of the treatment period between August 20 and August 26 was computed. Here, 333 players experienced at least one play break during these seven days. Of these 333 players, 163 wagered money at least once during the last seven days of the treatment period between September 9 and September 15 (48.9%). In the control period, 301 players deposited money at least on one day ten times or more between July 23 and July 29. Of these 301 players, 124 wagered at least once during the last seven days of the control period between August 12 and August 18 (41.2%). The difference of 7.7% between the 48.9% and the 41.2% who deposited at the end of the control and treatment period was statistically significant (Z=-1.958, *p* = 0.05).

## Discussion

The present real-world study investigated the effect of a forced online gambling session termination after ten deposits on a single calendar day for 60 min at a gaming operator’s online casino websites. The efficacy of the 60-minute mandatory play break on depositing/wagering money on the same day as well as the next day was evaluated. Furthermore, the impact on behavior several weeks in the future as well as the impact on player loyalty was also studied.

The authors were given access to 27 days of player data prior to the introduction of mandatory play breaks and 27 days of player data after the mandatory play break was introduced. Gambling activity was not evenly distributed on the gaming operator’s websites and for that reason, exactly the same number of weekdays was chosen for the control period and treatment period. There were no significant differences with respect to the distribution of the amount of money deposited and amount of money wagered before the tenth deposit between the control period and the treatment period. The same holds true with respect to gender and age (i.e., players gambling during the control period were not significantly older or younger compared to players gambling during the treatment period). The percentage of females was also not significantly different. Therefore, it was assumed that there were no major relevant demographic differences between the control period and the treatment period which could have had an impact on player behavior, other than the mandatory play break.

On average during the control period, 73% of players deposited at least eleven times during a single day from those players who deposited money at least ten times during a single day. After the mandatory play break was introduced, on average only 32% of players deposited money at least eleven times during a single day from those players who deposited at least ten times during a single day. This means that the percentage of players who stopped depositing money as a consequence of the mandatory play break rose from 27% to 68% on the day of the play break. This finding supports the hypothesis that mandatory play breaks not only interrupt a gambling session, but possibly brings to an end a state of dissociation if the player was experiencing one (Griffiths et al., 2006; Monaghan, 2009). A similar decrease in gambling activity was observed with respect to wagering money after the mandatory play break. After the mandatory play break was over, 55% of players wagered money at least once more during the rest of the day. Before the mandatory play break was introduced almost all of the players wagered money at least once after the mandatory play break (99.9%). This means that 45% of players stopped wagering money on that calendar day after receiving the mandatory play break.

The study also investigated the impact of the mandatory play break on the depositing and wagering of money during the following day. Findings indicated there were no significant differences in the control period and the treatment period with respect to the percentage of players who deposited during the next day following the mandatory play break. However, players in the treatment period were more likely to wager during the next day following a mandatory play break compared to players in the control period. This indicates that a mandatory play break for 60 min after ten deposits does not impact depositing the following day. However, the play break appears to increase the propensity to wager.

Blaszczynski et al. (2015) reported increased self-reported craving after longer mandatory play breaks in an experimental study Auer et al. (2019a) reported increased wagering after forced VLT session terminations in a real-world study. The present study investigated whether players with high losses were more likely to deposit and wager after the mandatory play break was over. Previous studies (i.e., Challet-Bouju et al., 2020; Perrot et al., 2018) have operationalized chasing losses using the metric of frequent depositing (which was also used in the present study). Chasing losses has been described as an important risk factor for problem gambling in several studies (e.g., Campbell-Meiklejohn et al., 2008; Lesieur, 1979). The present study analyzed the influence of the amount lost before a mandatory play break on the propensity to deposit and wager afterwards. The results in the present study do not support chasing losses as operationalized because there was no significant difference regarding the propensity to deposit money after the mandatory play break with respect to the amount of money lost before the mandatory play break. However, it should be noted that chasing losses using account-based tracking data not have a fixed definition.

**Fig. 4:**
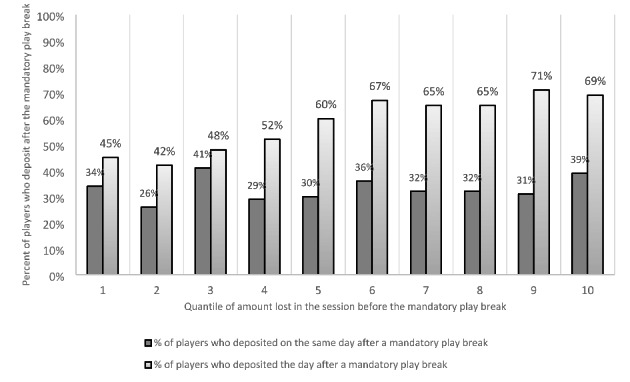
Percentage of players who deposited on the same day/next day after a mandatory play break during the treatment period. The ten groups on the x-axis are based on the amount lost in the session before the mandatory play break. Group 10 contains players who won (amount won larger than amount wagered)

The authors also analyzed the propensity to deposit money on the following day after a mandatory play break. The results indicated that there was a negative association between the amount of money won and depositing money the next day. Figure 4 shows that the percentage of players who deposited money the day after a mandatory play break increased as a function of the group they were assigned to on the x-axis. A larger number indicates a lower amount of money lost and Group 10 comprises the players who won money in the session immediately before the mandatory play break. This appears to indicate that players who lose less money in the session immediately before the mandatory play break are more likely to deposit money the next day. Players who won money in the session immediately before the mandatory play break were also more likely to deposit money on the next day compared to the 10% of players who lost the most (Group 1). Players who had lost less money or won more money than they wagered were more likely to deposit money the day following a mandatory play break. This perhaps indicates that players chase after their winnings rather than their losses. Such a finding has not been described or reported in any previous research.

The authors also wanted to understand which behavioral indicators were predictive of depositing on the same day after a mandatory play break. The independent variables were based on the session immediately before the mandatory play break (i.e., the amount of money deposited in the session immediately before the mandatory play break) and metrics computed for the time between midnight until the mandatory play break (i.e., the amount of money deposited between midnight and the mandatory play break). Results indicated that only 2.3% of the variance was explained by the 21 variables (see Table 1). This means that 97.7% of the variance is unaccounted for and the decision to deposit money again after the 60-minute mandatory play break is mostly accounted for by other factors which were not examined in the present study. Only three variables had a *p*-value smaller then 0.05. Firstly, players whose sessions were terminated between 01:00am and 05:00am in the morning were more likely to deposit at least once more during the remainder of the day. This is most likely related to the fact that there are still between 19 and 23 h left in the day for those players to deposit money. Secondly, the amount of money won during the session before the mandatory play break had a negative association with the propensity to deposit money at least once more in a single calendar day.

**Table 1:** Outcome of logistic regression with depositing after the mandatory play break as dependent variable. The number of stars indicate the size of *p*-values, *: *p* < 0.05. None of the variables are significant after Bonferroni correction

Variables	b	std err	z	*p*>|z|	sig
Intercept	-2.065700	0.887	-2.329	0.020	
Night session	0.537100	0.232	2.310	0.021	*
Session length	-0.003500	0.002	-1.461	0.144	
Amount of money deposited in the session	-0.002200	0.001	-1.720	0.085	
Amount of money withdrawn in the session	0.002900	0.002	1.915	0.055	
Number of money deposits in the session	0.030500	0.030	1.028	0.304	
Amount of money wagered in the session	0.000600	0.000	1.851	0.064	
Amount of money won in the session	-0.000800	0.000	-1.987	0.047	*
Number of games in the session	0.000054	0.000	0.235	0.814	
Amount of money lost in the session	-0.001400	0.001	-1.941	0.052	
Maximum monetary balance in the session	0.001600	0.001	2.628	0.009	*
Monetary balance at the start of the session	-0.003000	0.002	-1.595	0.111	
Monetary balance at the end of the session	0.001200	0.002	0.668	0.504	
Amount of money wagered on the last game in the session	-0.000400	0.002	-0.179	0.858	
Amount of money wagered on the first game in the session	0.011000	0.008	1.403	0.160	
Amount of money wagered all day before	0.000006	0.000	0.064	0.949	
Amount of money won all day before	-0.000012	0.000	-0.136	0.892	
Amount of money lost all day before	-0.000018	0.000	-0.106	0.915	
Amount of money deposited all day before	-0.000400	0.001	-0.794	0.427	
Number of money deposits all day before	0.145400	0.085	1.711	0.087	
Age	-0.001700	0.005	-0.324	0.746	
Female	-0.036300	0.114	-0.319	0.749	

Thirdly, it appears that the amount of money in the player’s gambling account (commonly referred to as the balance) before the mandatory play break had a positive association with the propensity to deposit at least once more on the same day (i.e., the larger the balance, the higher the propensity to deposit more money on the same day). The authors computed three variables based on the balance (the amount of money the player had in the gambling account at the start of the session, the amount of money the player had in the gambling account at the end of the session, the maximum amount of money the player had in the gambling account at any point of time during the whole session). The only statistically significant variable among those three was the maximum amount of money a player had in the gambling account in the session immediately before the mandatory play break. The larger the value, the larger the likelihood in depositing money once more during the remainder of the calendar day.

Although the results indicated that there was no significant effect of the mandatory play break in depositing money and wagering money the next day, the study also investigated longer-term effects. Depositing and wagering money during the last days in the treatment period was investigated among players who had received at least one mandatory play break during the first seven days of the treatment period. Comparisons of depositing and wagering money before the tenth deposit between control period and treatment period supported the notion that there were no significant differences, other than the introduction of the mandatory play break. The analysis did not show any significant increases or decreases of depositing or wagering money in the three weeks following players receiving a mandatory play break.

The introduction of a mandatory play break could potentially annoy players and consequently motivate them to leave an operator’s site and continue gambling elsewhere. As far as the authors are aware, mandatory play breaks for 60 min are not common among online casinos and after receiving them, players could simply look for less socially responsible online casinos that do not have mandatory breaks. Auer et al.(2019b) found that players who voluntarily set themselves limits were more likely to play with an operator after one year compared to players who did not set themselves voluntary limits. The present study computed the percentage of players who wagered at least once in the last seven days of the treatment period. The baseline were the players who received at least one mandatory play break during the first seven days of the treatment period. Two-fifths of those players (41.2%) wagered during the last seven days of the treatment period. In the control period, almost half of of players (48.9%) who deposited money at least ten times at on one occasion during the first seven days, wagered money during the last seven days of the treatment period. This difference was statistically significant and indicated a 7.7% decrease regarding the percentage of active players. The results appear to indicate that the 60-minute mandatory play break had a negative impact on loyalty. It should be noted that the study compared the number of players who deposited at the end of the treatment period to the number of players who deposited at the end of the control period. Both numbers are a consequence of various factors. One of them was assumed to be due to the mandatory play break, the other one due to the natural attrition rate. The attrition rate was therefore implicitly taken into account.

There were 1,461 events in the control period and 1,533 in the treatment period. It cannot be expected that the numbers are exactly the same as this was data from a real gambling platform in real time. Moreover, the study used a 27-day period not a full month but there is a trend throughout the month from the first day of the month to the last day of the month with respect to activity. This may be one reason for the slightly higher number of observed events in the treatment period. The present authors made a decision as to which periodicity (month or weekdays) were used to balance between the control period and treatment period. Because the variation across weekdays was larger, weekdays were chosen. The main hypothesis tested the effect of the mandatory play break on depositing/wagering on the day of the play break which is unlikely to be affected by the differences in the variation across the days.

The present study has a number of limitations that should be taken into account when interpreting the findings. First, only a relatively small number of participants actually received mandatory play breaks. Second, compared to other studies using behavioral tracking research, the overall sample size was modest. Third, more than one person might have been playing on the account although the number of shared accounts is likely to be few. Fourth, the data were from only from one gaming operator and may not be representative of online gamblers more generally. Fifth, the data were all collected during a relatively short time period (i.e., 54 days). Finally, all the data were from one nationality (British) and there may be cultural differences between online gamblers. To overcome and confirm the findings here, studies should replicate the study here utilizing data from other online gambling operators. Future studies should also include bigger sample sizes and examine longer periods of gambling activity.

## Conclusions

The findings of the present study demonstrated that a 60-minute mandatory play break impacts players’ depositing and wagering beyond the 60 min during which they cannot deposit or wager. A large percentage of players (41%) stop depositing for the remainder of the player day and a similar larger percentage of players (44.9%) stop wagering for the remainder of the day. This means that a mandatory play break in an online casino setting seems to prevent overspending during a short period of time. The results do not support previous assumptions and findings that mandatory play breaks lead to increased gambling afterwards. The present study was based on real-world data from actual players. Online casino operators could implement similar mandatory play breaks based on this study’s findings and regulators could use the insights to draft future regulatory requirements. The results also demonstrate that a 60-minute play break did not impact gambling on the next day and over a period of several weeks. Mandatory play breaks do not seem to be a player protection tool which changes behavior over a longer period of time. Future studies should investigate mandatory play breaks together with other player protection tools such as behavioral feedback, limit-setting and self-exclusion. Furthermore, the impact of mandatory play breaks on player loyalty should be studied. The latter is important for the adoption of mandatory play breaks by online casino operators.

## Appendix 1: List of variables and their corresponding definitions

**Table:**

Variable	Description
Age	Age in years
Gender	1 = Female, 0 = Male
Session length	Session length in minutes
Night session	Indicates whether the session happened between 01:00 AM and 05:00 AM
Amount of money deposited in the session	The amount of money deposited in the session
Amount of money withdrawn in the session	The amount of money withdrawn in the session
Amount of money wagered in the session	The amount of money wagered in the session
Amount of money won in the session	The amount of money won in the session
Amount of money lost in the session	The amount of money lost is defined as amount of money won minus amount of money wagered
Number of monetary deposits in the session	The number of deposits in the session
Number of games in the session	The number of games played in the session
Amount of money wagered on the first game in the session	The amount of money wagered in the first game in the session
Amount of money wagered on the last game in the session	The amount of money wagered in the last game in the session
Monetary balance at the start of the session	The amount of money in the account at the first game of the session
Monetary balance at the end of the session	The amount of money in the account in the last game of the session
Maximum monetary balance in the session	The maximum amount of money which was in the account during the session
First balance in the session	The monetary balance of the gambling account at the beginning of the session
Last balance in the session	The monetary balance of the gambling account at the end of the session
First amount of money wagered in the session	The amount of money wagered on the first game in the session
Last amount of money wagered in the session	The amount of money wagered on the last game in the session
Amount deposit all day before	The amount of money deposited on that calendar day up to the tenth deposit
Amount of money wagered all day before	The amount of money wagered on that calendar day up to the tenth deposit
Number of games all day before	The number of games played on that calendar day up to the tenth deposit
Amount of money deposited all day after	The amount deposited after the tenth deposit up to 24 h
Amount of money wagered all day after	The amount of money wagered after the tenth deposit up to 24 h
Amount of money deposited next day	The amount of money deposited on the next calendar day
Amount of money wagered next day	The amount of money wagered on the next calendar day
